# Aggression, Alexithymia and Sense of Coherence in a Sample of Schizophrenic Outpatients

**DOI:** 10.3390/healthcare10061078

**Published:** 2022-06-10

**Authors:** Argyro Pachi, Athanasios Tselebis, Ioannis Ilias, Effrosyni Tsomaka, Styliani Maria Papageorgiou, Spyros Baras, Evgenia Kavouria, Konstantinos Giotakis

**Affiliations:** 1 Psychiatric Department, “Sotiria” General Hospital of Chest Diseases, 11527 Athens, Greece; irapah67@otenet.gr (A.P.); tsomaka@gmail.com (E.T.); stellamar4@yahoo.gr (S.M.P.); spyrosbaras@gmail.com (S.B.); e.kavourgia@gmail.com (E.K.); cgiotakis@gmail.com (K.G.); 2 Department of Endocrinology, “Elena Venizelou” Hospital, 11521 Athens, Greece; iiliasmd@yahoo.com

**Keywords:** aggression, alexithymia, sense of coherence, schizophrenia, comprehensibility

## Abstract

Schizophrenia elevates the risk for aggressive behavior, and there is a need to better understand the associated variables predicting aggression for treatment and prevention purposes. The aim of the present study is to determine the relationship between alexithymia, sense of coherence and aggressive behavior in a sample of schizophrenic outpatients. Using a correlational research design, standardized self-report questionnaires assessed aggression (brief aggression questionnaire—BAQ), alexithymia (Toronto Alexithymia Scale—TAS) and sense of coherence (sense of coherence questionnaire—SOC) in a sample of 100 schizophrenic outpatients in clinical remission. Participants reported high levels of aggression and alexithymia along with reduced sense of coherence. Significant negative correlations were evidenced among scores on the SOC scale (*p* < 0.001) with both the TAS as well as with the BAQ scales. However, a positive correlation (*p* < 0.001) was observed between the TAS and BAQ scales. Regression indicated that 27% of the variation in the BAQ rating was explained by the TAS, while an additional 17.8% was explained by the sense of coherence. The difficulty identifying feelings of alexithymia and the comprehensibility and manageability components of sense of coherence significantly predicted anger, hostility and physical aggression. Sense of coherence mediated the relationship between alexithymia and aggression. From the path analysis, comprehensibility emerged as the key factor counterbalancing alexithymic traits and aggressive behaviors, and manageability effectuated higher anger control. The findings hold practical implications for the treatment and rehabilitation of schizophrenic patients.

## 1. Introduction

Most researchers agree on the presence of an increased risk of aggressive behavior in patients with schizophrenia, but there is considerable heterogeneity in the reported rates of such aggression and uncertainty as to the causes of this heterogeneity [1,2]. Predisposing factors—namely genotype; prenatal and perinatal insults; early adversity as in childhood maltreatment; conduct disorders; comorbid antisocial personality disorder/psychopathy; and precipitating factors, in particular emergence of psychotic symptoms, neurocognitive impairment, substance abuse, nonadherence to treatment and stressful experiences in adult life—can result in risk interactions, increasing the likelihood for the emergence of aggressive behavior [3,4,5]. In particular, comorbidity with substance abuse increases the incidence of aggressive behavior in patients with schizophrenia with personality traits and social factors probably mediating the relationship between substance abuse and aggressive behavior in these patients [6,7,8,9]. Medication nonadherence may also serve as a contributing factor, particularly if it precedes substance abuse [10].

The literature reports differences in brain structure and function associated with aggression in schizophrenia, particularly in areas involved in the formation of psychosis symptoms and affective regulation. The most consistent findings from the structural studies were reduced volumes of the hippocampus and the frontal lobe (i.e., the orbitofrontal and anterior cingulate cortex), and functional studies mainly showed variations in the frontal lobe and amygdala [11,12]. As hypothesized, volume reductions in the hippocampus may predispose individuals with schizophrenia to be less sensitive to social and emotional cues, which might give rise to conflicts and the inability to perceive signals for solutions, leading to conflict escalation [13]. Additionally, functional and neurophysiological studies evidenced an inefficient integration of the information by the dorsal anterior cingulate, between the frontal and limbic regions, in schizophrenia patients with a history of violence during negative emotion processing [14,15]. The anterior cingulate plays a central role in processes that are critical to successful emotion regulation, conflict and performance monitoring, as well as emotional awareness [16,17]. Aberrant dorsal anterior cingulate functional connectivity patterns are consistent with impaired cognitive control over emotions [14]. Aggressive patients display strong reactivity to negative stimuli, which may interfere with response inhibition and lead to impulsive aggression. Relevant research suggested that psychotic symptoms in schizophrenia patients preceded a violent incident only in a small percentage of cases, supporting the idea that behavioral disinhibition and emotional dysregulation are important factors for aggressive behavior in patients with schizophrenia [18].

Aggression in schizophrenia can occur at any time during the disease’s course, has significant implications for patient care and treatment and raises the risk of harm. In search for a potential biological signal for early assessing aggressive risk in schizophrenic patients, recent studies identified increased inflammation (CRP levels, leukocyte count and neutrophil to lymphocyte ratio) as a potential biological correlate of aggression [19,20]. The presence of aggressive behavior in schizophrenic patients indicates the severity of the disorder to some degree, and the level of inflammation decreases as the disease goes into remission. The disruptive effect of early-life stress on the immune system is partly involved in brain mechanisms that regulate aggressive behavior in schizophrenia patients, suggesting a link of clinical significance.

A more dynamic feature which has been posited as having an important role in the pathway to aggression is alexithymia [21,22]. Alexithymia is a mental condition characterized by difficulties identifying and describing one’s own feelings, externally oriented thinking, and limited imaginative capacity [23]. The majority of research has approached alexithymia as a stable personality trait, thought to be developmental in nature, reflecting a lack in emotion regulation and cognitive processing [24]. Awareness of one’s own emotions can prevent us from primitive, uncontrolled emotional responses when facing negative events. Given their inability to identify, manage and express their true emotions, individuals who are alexithymic exhibit high levels of anger and more aggressive behavior. Research indicates that it was primarily the difficulties with identifying feelings aspect of alexithymia that was related to aggression [25]. Brain imaging studies on alexithymia displayed impaired cognitive emotional processing, and—owing to this impairment—alexithymics experience inflexible cognitive regulation. Additionally, they showed weak responses in structures necessary for the representation of emotion used in conscious cognition and stronger responses at levels focused on action [26]. 

Meta-analyses of functional and structural brain imaging studies have identified the amygdala, the insula, the anterior cingulate cortex and regions of the prefrontal cortex as key correlates of alexithymia in the brain [27,28,29]. Interestingly, alexithymia is commonly associated with abnormalities of both the anterior cingulate cortex and the insula [30], and impairments in these regions—as suggested—may contribute to aggression, particularly reactive aggression [31]. Contemplating the nature of the alexithymia–aggression relationship, other research evidenced increased right amygdala volume as a common neurobiological denominator for both alexithymia and reactive aggression [31]. Another possible explanation for the association between alexithymia and aggression concerns difficulty labeling emotions. In neuroimaging studies, when labeling emotions, the prefrontal cortex is engaged, while activity in the amygdala is simultaneously reduced, indicating that the cognitive act of labeling emotions dampens the emotional response [32].

Studies have described a variety of deficits in emotion processing in individuals with schizophrenia and identified dysfunction in the domains of emotion expression, emotion experience and emotion recognition [33,34]. Consequently, individuals with schizophrenia who are unable to accurately recognize emotional expressions face problems of adaptability in social life. Inability to decode the social cues projected by others, in which emotions are contained, can lead to misinterpretation of the ambiguous signals received, violation of personal boundaries and possible manifestation of inappropriate or even aggressive behavior. In addition, people with schizophrenia also experience problems in identifying and expressing their own emotions. One of the key areas in which they fall short which is directly related to the expression of their emotional state is that of communication. Although they experience emotions, they often find it difficult to describe and express them in words, sometimes giving the impression that they feel nothing. Emotional dysregulation is closely linked to aggression. Individuals who are unable to express their emotional state experience an uncomfortable, unpleasant and uncontrollable situation that is difficult to tolerate, and thus resort to the use of aggression more easily in order to either communicate this unpleasant experience or to avoid it.

Over recent years, research has indicated that schizophrenia patients are also likely to have reduced empathic ability when interacting with others in everyday life and are less accurate at tracking the positive and negative affective state of another person [35]. These deficits tend to cover every aspect of empathy, from the cognitive to the emotional dimension [36]. Two other concepts closely related to empathy that are compromised in schizophrenia are theory of mind and insight. People with schizophrenia have difficulty processing both their own mental state and those of others, with the result that they are less able to interpret and predict others’ behavior. Deficits in the above characteristics can lead to socially dysfunctional and aggressive behaviors. Conversely, understanding another person’s emotional and mental state can act as a deterrent to the occurrence of dysfunctional behavior.

Scientific studies support that alexithymia is highly prevalent among schizophrenic patients [37,38,39]. Whether it is a trait characteristic in deficit patients and a state related to many symptoms, such as flattening of affect, poverty of speech, depression and anxiety in nondeficit patients, or being a separate construct related to dysfunctions in both cognitive and affective processes remains a matter of controversy. Several authors suggested that alexithymia may be a vulnerability factor for the development of schizophrenia and, more specifically, may be an underlying cause of social dysfunction [40,41]. Various core aspects of social cognition have been found to be disrupted in schizophrenia, including emotion recognition [42] and theory of mind [43,44]. Much of the broader social cognitive literature in schizophrenia has focused on the ability to understand the affective states of others, such as via emotion recognition (the ability to decode affective cues) or theory of mind (the ability to understand another’s beliefs and desires). In contrast, alexithymia primarily refers to the ability to understand one’s own affective experience and therefore seems conceptually closer not only to the construct of emotion regulation, but also to affective empathy (one’s emotional response to the cognitive or affective state of another). Neuroimaging studies investigating pathology underlying alexithymia in schizophrenia report gray matter alterations of the left supramarginal gyrus and reduced white matter integrity within the corpus callosum, mostly in the left part of the superior and inferior longitudinal fasciculi, the inferior occipitofrontal fasciculus, the anterior and posterior thalamic radiation and the precuneus white matter [45,46]. 

Alexithymia has been shown to be associated with both detachment from the self and inadequate differentiation between self and other [47], both of which are included in schizophrenia-spectrum psychopathology. In the same way that the fragmentation of the self may lead to psychotic phenomena, it may also result in impaired ability to differentiate and express one’s own emotional experience. Phenomenologically-oriented researchers propose that a disturbance of the basic sense of self is at the clinical core of the schizophrenia spectrum [48]. These abnormal experiences, possibly driven by neurocognitive disturbances, may evolve into first-rank psychotic symptoms [49]. Low baseline levels of basic self-disturbances and further reductions over time independently predict recovery [50]. Significant association between basic self-disturbances and sense of coherence, not mediated by other clinical symptoms, was reported, identifying high levels of basic self-disturbances as independent contributors to poor sense of coherence [51].

Sense of coherence (SOC) was proposed by Antononsky as a construct that expresses the degree to which a person has a diffuse and dynamic, but lasting sense that the internal and external stimuli and stressors in their environment are understandable (i.e., predictable, structured and explicable), manageable (i.e., there are resources available to meet the requirements of these stimuli) and meaningful (i.e., the requirements are challenges that are worth committing to and addressing) [52]. The SOC is often considered to be a stable entity that develops in young adulthood and stabilizes around the age of 30, and as a personality trait it, is more likely to be a predictor of behavior [53,54]. In searching for a relationship between sense of coherence and aggression, a low level of coherence (perceiving stimuli as threatening accompanied by a lack of sufficient internal and external resources to effectively solve problems) may manifest in aggressiveness in the affective and cognitive dimensions (anger, hostility) and also in the instrumental parts of aggressive behavior (verbal, physical aggression) [55].

Sense of coherence and alexithymia exert opposite influences as to the treatment of psychological and physiological disorders, effectuating positive and negative consequences, respectively [56,57]. With reference to relevant evidence, alexithymia may lead to adverse health outcomes as a result of emotion dysregulation and unsuccessful stress and anxiety management, while sense of coherence is regarded as a protective factor that promotes recovery, allowing a person to be resilient in the face of challenges [58,59]. Individuals with a high sense of coherence are likely to perceive stressors as explicable, have confidence in their coping abilities, and feel engaged and motivated to cope with stressors [60]. Inversely, individuals with alexithymia have lower levels of physical functioning, less energy, poorer emotional wellbeing, poorer social functioning and poorer general health [61]. The salutogenesis theory recognizes sense of coherence as a key component of health, whereas alexithymia is presumed to play an important predisposing role in the pathogenesis of diseases. Strengthening the sense of coherence and coping is conducive to recovery [62,63].

According to the salutogenetic approach to the problem of health and disease, higher sense of coherence protects people from the onset of disorders and, if they emerge, aids in accelerating the recovery of health [64,65]. Major mental illnesses, like schizophrenia, are usually expected to run a chronic course with varying trajectories, sometimes in the form of a steady or gradual deterioration and other times with improvements and acute exacerbations with unpredictable effects on outcome. Prognosis varies on a continuum between satisfactory recovery and total disability; although, according to follow-up studies, several schizophrenic patients have a more favorable course outcome [66,67]. Research indicates that people with schizophrenia with a high sense of coherence experienced less severe psychopathological symptoms and a higher overall level of function while also obtaining better results in treatment [68,69].

Over the past decades, evidence of the association between schizophrenia and aggression has accumulated, thereby identifying a multitude of relevant risk factors [70,71,72]. The presence of alexithymia among patients with schizophrenia has been extensively studied, and heightened levels of alexithymia in a number of different schizophrenia samples have been evidenced [38,41,73,74]. However, studies focused on how alexithymia may give rise to aggression in patients with schizophrenia are scarce [21,75]. The relationship between alexithymia and aggression has also been investigated mostly in community samples, mixed psychiatric and substance dependence inpatients, adolescents, violent offenders and forensic patients [29,76,77,78,79,80,81,82,83,84,85]. The association between sense of coherence and aggression has been investigated among juveniles from reformatories, but also among female employees and coronary heart disease patients, as predictors of health-related quality of life [55,86,87,88]. Sense of coherence in schizophrenia and among delusional patients has been studied in relation to psychopathology and in order to predict remission and risk of relapse [68,89,90,91,92]. Finally, the relationship between alexithymia and sense of coherence has been investigated among university students, patients suffering from fibromyalgia and among attention deficit hyperactivity disorder patients [56,58,93].

Schizophrenia-related aggression poses a severe threat to the patient’s and society’s safety and necessitates the development of interventions with specific or nonspecific anti-aggressive properties. There are various treatment choices apart from pharmacological treatments for addressing aggression in schizophrenia patients. Psychological treatments and other nonpharmacological interventions may be of interest when the etiology of aggression is not a target for pharmacological agents. Elucidating the role of alexithymia on aggression in schizophrenia suggests new modes of treatment which would target these specific underlying impairments. A review examining the effects of psychological interventions on alexithymia concluded that it is partly modifiable with these therapeutic interventions, offering suggestions for future research [94]. Similarly, salutogenic-based approaches offer promising results, strengthening sense of coherence and effectuating positive outcomes in key variables for personal recovery in people with schizophrenia [95,96].

The exploratory purpose of this study was to investigate the possible association between aggression, alexithymia and sense of coherence in a sample of schizophrenic outpatients since there is no study in the literature that simultaneously examines the relationship of these variables. We argue that specific components of sense of coherence, as well as alexithymic traits involving emotional dysregulation, offer insight into schizophrenic outpatients’ aggressiveness influencing their self-reported levels of aggression. 

The specific aim of this study is to verify whether certain alexithymia and sense of coherence dimensions serve as mediators predicting various aspects of aggression. Differently, considering that sense of coherence is related to the ability to regulate and manage emotions appropriately [87], counterbalancing the limited ability of alexithymic individuals, we aimed to investigate the intervening role of sense of coherence in the relation between alexithymia and aggression. Results would provide a rationale for the development of psychological interventions [75,97] specifically targeted at improving alexithymia and sense of coherence in outpatients with schizophrenia in order to control their aggressive tendencies and cope with their aggressive feelings themselves.

## 2. Subjects and Methods

### 2.1. Research Design

In this study, a correlational research design was used. It was conducted with outpatients treated at the Outpatient Psychiatric Department of Sotiria General Hospital between September 2021 and February 2022 after approval from the Clinical Research Ethics Committee of Sotiria General Hospital (Number 24252/27-9-21). According to ethical considerations, participation in the survey was completely voluntary. First, the researchers explained the research objectives and patients were assured that the information would remain confidential. After each participant was informed about the study, they provided written and verbal informed consent. Once recruited, all participants were asked to answer to a semi-structured form designed by research staff to collect demographic data and to fill a battery of self-report questionnaires to assess their self-reported levels of aggression, alexithymia and sense of coherence. At the end, all of the responses were collected anonymously. 

### 2.2. Study Participants

Adopting purposive sampling, the study involved 100 consecutive outpatients with confirmed psychiatric diagnoses of schizophrenia, using the International Classification of Diseases-10 (ICD-10) coding system, who attended the Psychiatry Outpatients Department for maintenance treatment. Eligibility criteria included: (i) aged between 18 and 65 years old; (ii) being in clinical remission in a post-acute phase of illness as defined by no hospitalizations and no changes in psychotropic medication or psychosocial status within 30 days prior to enrollment; (iii) with a history of at least two prior psychiatric hospitalizations (greater diagnostic confidence in confirming schizophrenic disorders), but not more than five hospitalizations (to exclude patients with residual schizophrenia); (iv) coherent verbal contact during the filling of data collection form. Exclusion criteria for participants were psychotic disorders related to clinical medical conditions or substance use; substance addiction and history of substance use in the last six months; uncorrected visual or hearing impairments; neurological disorders or damage to the central nervous system; developmental disability; and signs of intellectual disability, severe cognitive and neuropsychological impairment, personality disorders or schizoid and schizotypal personality traits, other psychiatric comorbidities, namely social anxiety disorder and a record of current substance or alcohol abuse. The majority of participants (68%) were on atypical antipsychotic monotherapy with confirmed antiaggressive properties, while the rest of them were additionally treated with a combination of adjunctive anticonvulsants. A comprehensive health assessment and clinical evaluation of substance abuse were conducted upon study enrollment. 

### 2.3. Minimal Sample Size Calculation

A sample size of 100 was deemed adequate given five independent variables (IVs) to be included in the hierarchical linear regression analysis (N > 50 + 8 m, N = number of Participants and m = number of IVs) [98]. To confirm sample size adequacy, a post hoc power analysis was carried out using G-Power software [99]. The calculation indicated that with a sample size of 100, effect size f^2^ = 0.8116 (derived from the R^2^ = 0.448), an alpha of 0.05 and five predictors, an excellent power of 1.00 was identified. The same procedure was followed to verify sample adequacy for the other hierarchical linear regression analyses models built with six predictors. Additionally, a Monte Carlo power analysis for indirect effects was performed through an online application [100]. The results show that a power of 0.95 with a confidence level of 99% is reached with only 60 participants in a simple mediation model. Finally, according to the rule of thumb recommended by Kline [101], an adequate sample size should be 10 times the number of the parameters in path analysis (six parameters were involved in the research path analysis). 

### 2.4. Measurement Tools

Demographic and social data from study participants included age, gender and years of education. 

### 2.5. Brief Aggression Questionnaire

The brief aggression questionnaire (BAQ) is a 12-item self-report measure of aggression. The questionnaire asks participants to rate on a scale from 1 (strongly agree) to 5 (strongly disagree) the degree to which statements describing behaviors and emotions, are characteristic of themselves. The ΒAQ measures aggression in the domains of physical aggression, verbal aggression, anger, and hostility. The total aggression score was calculated by summing these four factors’ scores. Higher scores indicate higher levels of aggression. The questionnaire was translated and back translated, from English to Greek and vice versa, by three bilingual translators and adapted in Greek population [102]. BAQ has been proposed as a valid and reliable instrument (Webster et al., 2014) with adequate temporal stability and convergent validity with other behavioral measures of aggression [103]. Cronbach’s alpha in this study was 0.731.

### 2.6. Sense of Coherence Questionnaire

To measure sense of coherence, we used the short version of a self-rating scale, the sense of coherence questionnaire (SOC-13), developed by Antonovsky [52]. SOC-13 comprises of three components (a) a cognitive component, labeled comprehensibility, representing the ability to understand and integrate internal and external experiences, (b) an instrumental component, labeled manageability, representing the ability to handle challenges and cope with stressful situations and (c) a motivational component, labeled meaningfulness, representing the ability to make sense of experiences and view them as worthy challenges [52,104]. Responses to each question are given using a 7-point Likert scale ranging from 1 (“very common”) to 7 (“very rare or never”). Scores range from 13 to 91, with higher numeric values representing a higher degree of SOC. The short version of SOC-13 has been standardized in the Greek population and seems to be a reliable and valid instrument with a Cronbach alpha of 0.83 [104].

### 2.7. Toronto Alexithymia Scale

The Toronto Alexithymia Scale (TAS-20) is one of the most commonly used self-report measures of alexithymia [105]. It consists of 20 sentences and includes 3 subscales: emotion recognition, which measures the extent to which people report difficulty in identifying their own feelings (DIF); emotion expression, which measures the extent to which people report difficulty in describing feelings to others (DDF); and externally-oriented thinking (EOT), which measures the extent to which people report a tendency to focus on the concrete details of external events rather than of their own inner experience. The sentences are scored using a 5-point Likert scale from 1 (strongly disagree) to 5 (strongly agree) with total scores ranging from 20 to 100. The scale has good reliability and validity in both its original version and in the Greek adaptation [106] that was used in the present study. The distinctive cutoff scores to indicate the degree of alexithymic characteristics were as follows: ≤50 indicated no alexithymia, 51–60 indicated borderline alexithymia, and ≥61 indicated alexithymia [105]. The Cronbach’s alpha for the scale in this study was 0.809. 

### 2.8. Statistical Analysis

**Figure 1:**
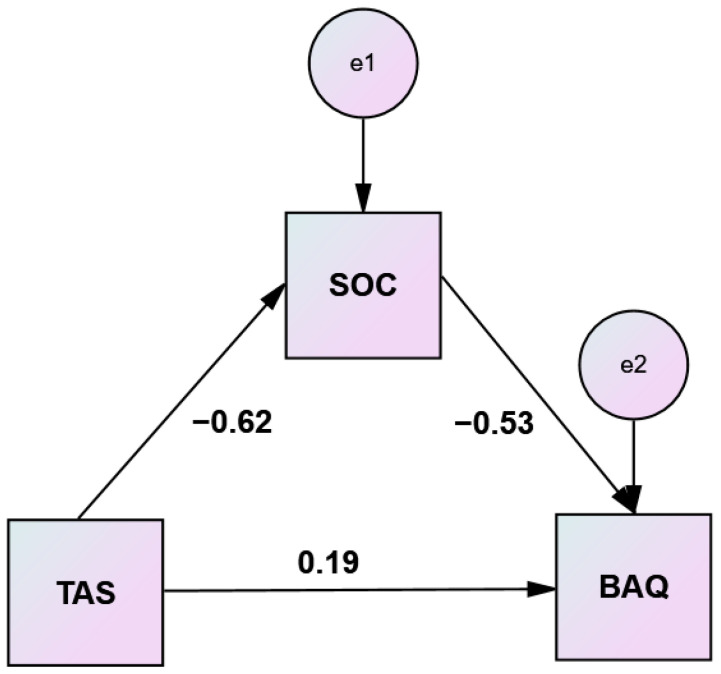
Simple mediation analysis of sense of coherence (SOC) on Toronto alexithymia scale (TAS)–brief aggression questionnaire (BAQ) relationship.

**Figure 2:**
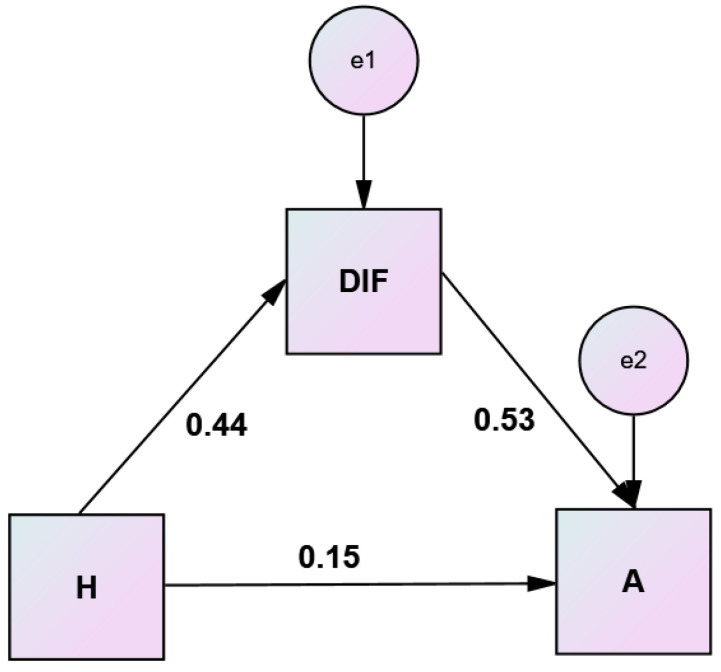
Simple mediation analysis of difficulty identifying feelings (DIF) on hostility (H)–anger (A) relationship.

**Figure 3:**
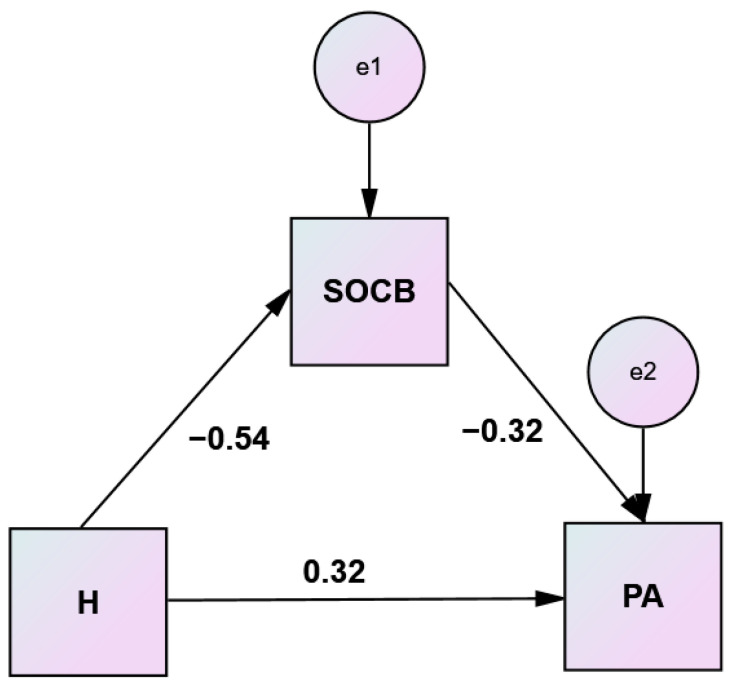
Simple mediation analysis of comprehensibility (SOC B) on hostility (H)–physical aggression (PA) relationship.

**Figure 4:**
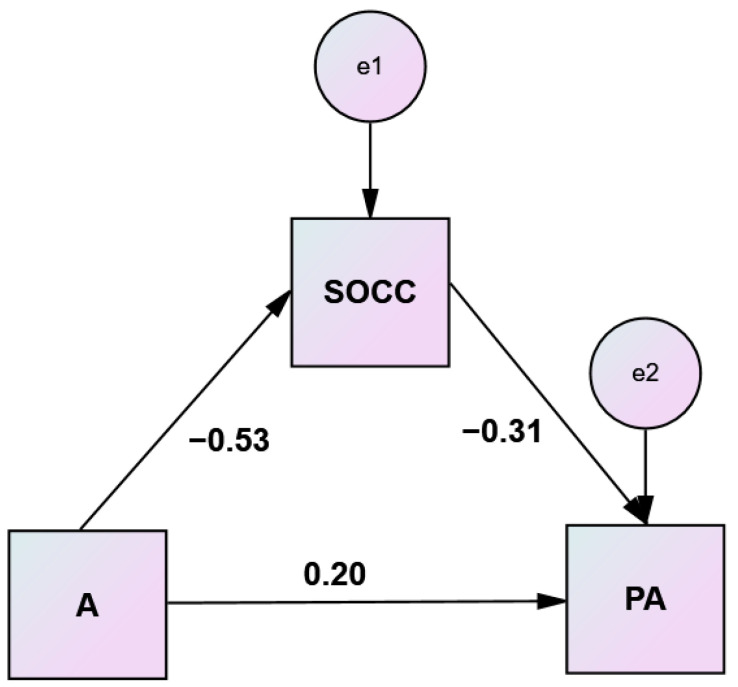
Simple mediation analysis of manageability (SOC C) on anger (A)–physical aggression (PA) relationship.

**Figure 5:**
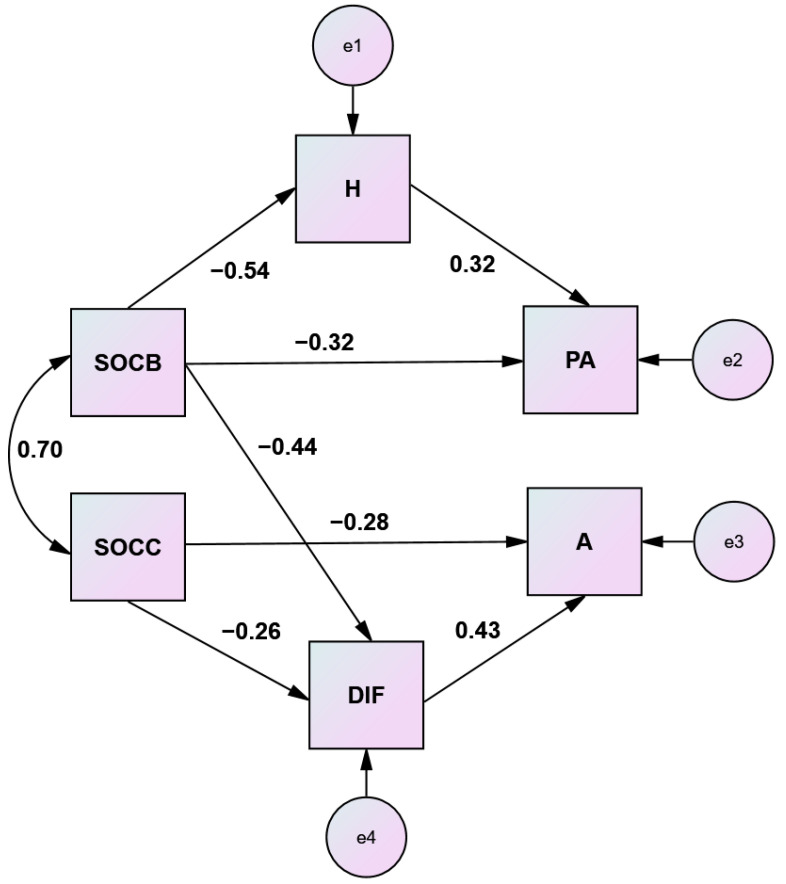
Path model illustrating patterns of effect within a system of research variables. Note: Standardized coefficients are presented.

Descriptive statistics were computed for all variables in the analysis. Independent sample *t*-tests assessed for gender differences. The prevalence of alexithymia was determined as a percentage. The internal consistency reliability of the BAQ, SOC-13 and TAS-20 in our sample was evaluated using Cronbach’s alpha coefficient (≥0.70). The Shapiro–Wilk test was used to assess the normality of the data. Pearson correlation was performed to determine the strength and direction of the relationship between variables. Hierarchical linear regression analyses were built to investigate whether related variables were significant predictors of aggression while controlling for other covariables. The assumption testing (linear relationship, independence, homoscedasticity and normality) was carried out by visual inspection of the variables, residuals and collinearity statistics and quantile–quantile (QQ) plots, probability–probability (PP) plots and scatterplots. A bootstrap approach was used to test the significance of the indirect effect of alexithymia on aggression through the mediating role of sense of coherence. The SPSS PROCESS Macro (Hayes, 2013) was used to conduct simple mediation analyses, computing 5000 bootstrap resampling with replacement from the original dataset to estimate 95% confidence intervals (CIs) for the indirect effects (CIs that do not include zero indicate a significant indirect effect). For the sake of parsimony, mediation models were run, including TAS-20 and SOC subscales, predicting each of the BAQ subscales. Path analysis was performed in order to concurrently examine the impact of a set of predictor variables (certain TAS-20 and SOC subscales derived from regression and mediation model results) on the BAQ subscales, which were handled as dependent variables and thus identify which are the most important (and significant) paths. This may have implications for the plausibility of our prespecified hypotheses. A structural model with observed variables was tested using a covariance matrix as input and maximum likelihood estimation. Assumptions (linearity, causal closure, unitary variables) were respected. Maximum likelihood estimation (MLE) indices were calculated in order to assess the correspondence of the model with the data: chi-square statistics, root mean square error of approximation (RMSEA) and comparative fit index (CFI). All *p* values were two-tailed, and the statistical significance level was set at *p* < 0.05. SPSS software, version 23, was used for the statistical analysis. SPSS AMOS 23 Graphics enabled the presentation of Figure 1, Figure 2, Figure 3, Figure 4 and Figure 5.

## 3. Results

### 3.1. General Characteristics of Participants and Scores on Outcome Variables

The study included 100 participants (45 men and 55 women). Means and standard deviations for general characteristics of participants and all key variables are presented in Table 1. The mean BAQ score was statistically higher compared to the corresponding score in the Greek general population [107], (30.93 vs. 23.22, sample *t*-test *p* < 0.01). A total of 22% of schizophrenic participants scored above the cutoff on the TAS scale. The mean TAS score was statistically higher compared to the corresponding score in the general population [61], (49.10 vs. 45.8, sample *t*-test *p* < 0.05), but comparable to the average score observed among patients with chronic somatic diseases (49.10 vs. 48.2 sample *t*-test *p* > 0.05) [108]. The average SOC was statistically lower compared to the mean score from the standardization studies [104] in the Greek general population (56.57 vs. 59.85 sample *t*-test *p* < 0.05). No statistically significant differences were observed between men and women as to the BAQ, TAS, or SOC scores or any of their subscales. Significantly higher BAQ and lower SOC scores were evidenced among alexithymic participants (36.59 ± 8.7 vs. 29.33 ± 7.42, t = 3.564, *p* = 0.001 and 45.95 ± 16.33 vs. 59.42 ± 1433, t = −3.44, *p* = 0.002, correspondingly). 

### 3.2. Correlations among Continuous Variables

Significant negative correlations were evidenced among scores on the SOC scale (*p* < 0.001) with both the TAS as well as with BAQ scales. However, a positive correlation (*p* < 0.001) was indicated between TAS and BAQ scales (Table 2). 

Results from correlations among subscales of the TAS, BAQ and SOC are presented in Table 3.

### 3.3. Hierarchical Linear Regression Analyses

A three-stage hierarchical linear regression analysis was conducted to evaluate the prediction of BAQ scores from the general characteristics of participants (age, gender and education), TAS scores and SOC scores. For the first block analysis, the predictor variables age, gender (coded as 1 = male, 2 = female) and education were analyzed. The results of the first block revealed the model not to be statistically significant (*p* > 0.05). For the second block analysis, the predictor variable TAS scores were added to the analysis and contributed significantly to the regression model, F (4, 92) = 8.501, *p* < 0.001) accounting for 27% of the variation in aggression. Introducing the SOC scores variable at stage three explained an additional 17.8% of variation in aggression and this change in R² was significant, F (5, 91) = 14.757, *p* < 0.001. Both the TAS scores and SOC scores were significant predictors of aggression. Together, the two independent variables accounted for 44.8% of the variance in aggression. Participants’ predicted aggression was equal to 37.84 + 0.108 (TAS) − 0.272 (SOC) (Table 4).

Two-stage hierarchical linear regression analyses were conducted to evaluate the prediction of BAQ and subscales scores from the scores on the TAS and SOC subscales. For the first block analysis, the predictor variables were the scores on TAS subscales. and for the second block analysis, the scores on the SOC subscales were added. In these analyses, DIF, SOC A, SOC B and SOC C predicted the dependent variables (Table 5).

### 3.4. Simple Mediation Analyses

To clarify the nature of the relationship between alexithymia and aggression and answer one of the research questions, we investigated the underlying mechanism by which alexithymia influences aggression through sense of coherence. The objective was to examine the impact of alexithymia on aggression as mediated by sense of coherence. It was hypothesized that being alexithymic will positively predict aggression. Additionally, it was hypothesized that sense of coherence will mediate this relationship. A simple mediation analysis, using the bootstrap method, was conducted to test these hypotheses. Analyzing the indirect effects, the results reveal that sense of coherence significantly mediated the relationship between alexithymia and aggression [(B 0.2093, 95% C.I. (0.1234, 0.2966), *p* < 0.05, Table 6]. Sense of coherence accounted for 65.55% of total effect. These findings provide some evidence that alexithymic patients are less likely to exhibit aggression provided they have high sense of coherence. Nevertheless, alexithymia still contributes to aggression beyond what is accounted for by sense of coherence. Standardized coefficients for the variables are depicted in Figure 1. 

A bootstrap approach was used to test the significance of the indirect effect of hostility on anger through the mediating role of difficulty identifying feelings. Results of this mediation analysis are displayed in Table 7 and illustrated in the Figure 2.

Results of the mediation analyses to test the significance of the indirect effects of hostility and anger on physical aggression through comprehensibility and manageability are displayed in Table 8 and Table 9 and illustrated in Figure 3 and Figure 4.

### 3.5. Path Analysis

Path analysis was used to determine the pathways by which the alexithymia and sense of coherence dimensions interact to influence modes of aggression. From regression and mediation analysis results, it was expected that comprehensibility and manageability would exert their protective effects, counteracting physical aggression and anger both directly and indirectly through hostility and the difficulty identifying feelings dimension of alexithymia. A structural model with observed variables was tested using a covariance matrix as input and maximum likelihood estimation. This type of analysis provides a comprehensive picture of the nature of the associations between the predictor and dependent variables of interest. Comprehensibility and manageability served as predictor variables and physical aggression and anger were treated as the dependent variables. Results suggested that the measures of fit of the model were satisfactory, indicating adequate fit (*p* = 0.325, CMIN/df = 1.156, comparative fit index CFI = 0.995, normed fit index NFI = 0.968, parsimonious normed fit index PNFI = 0.552, incremental fit index IFI = 0.996, Tucker–Lewis index TLI = 0.990 and the root mean square error of approximation RMSEA = 0.040). No modifications were necessary for the model. The nonexistence of an arrow between two variables means that these two variables are not significantly related. Figure 5 presents the set of hypotheses about the relations between the aforementioned variables. 

## 4. Discussion

The present study confirmed high levels of aggression and alexithymia and low levels of sense of coherence among schizophrenic outpatients. Participants with alexithymic characteristics reported higher aggressive tendencies and lower sense of coherence capacities. The observed correlations supported all the main assumptions of the relationships between the study variables. Alexithymia and sense of coherence accounted for 44.8% of the variance in disclosed aggression. The difficulty identifying feelings dimension of alexithymia and the comprehensibility and manageability components of sense of coherence significantly predicted anger, hostility and physical aggression. Sense of coherence mediated the relationship between alexithymia and aggression. The difficulty identifying feelings dimension of alexithymia mediated the relationship between hostility and anger. Comprehensibility mediated the relationship between hostility and physical aggression, and manageability mediated the relationship between anger and physical aggression. From the path analysis, comprehensibility emerged as the key factor counterbalancing alexithymic traits and aggressive behaviors, and manageability effectuated higher anger control. The main hypotheses were supported, indicating that high SOC scores predicted high physical aggression buffering and anger control, both directly and indirectly neutralizing alexithymic traits and hostility.

The presence of hostility in schizophrenic patients is not limited to the acute phase of the disease [109] but persists for a long time after hospitalization [110] and may be a predisposing factor for the emergence of anger and verbal or physical aggression. As opposed to enduring hostility, anger is a temporary but highly intense negative emotional state that usually abates but easily relapses in people who are temperamentally characterized by hostility due to their increased susceptibility to situations of anger [111]. Imaging studies aiming to clarify the neuroanatomical basis of hostility-related dimensions in schizophrenia patients who exhibited high levels of urgency, impulsivity and aggressiveness reported dysfunction of neuronal circuits involving the amygdala, striatum, prefrontal cortex, anterior cingulate cortex, insula and hippocampus [112].

The literature suggests that aggression and alexithymia are related to each other in the mentally ill [21]. Poor emotional awareness [33] and emotion dysregulation [113] are possible implicating mechanisms underlying aggression. By definition, alexithymia is considered to be a disorder of affect regulation [113], and there is evidence that some facets of emotion regulation may be disrupted in schizophrenia [114,115,116]. A possible etiological sequence is that limited emotional awareness interferes with emotion regulation, effectuating indirect consequences as to emotional response, thereby mediating aggression [117]. 

Awareness and understanding of emotions are common features of both alexithymia and emotion regulation. Research reports that alexithymics have a variety of emotion regulation difficulties and outline the nature of emotional dysfunction in alexithymia. Nonacceptance of emotional reactions, lack of emotional clarity, difficulties with goal-directed behavior and impulse control, as well as limited access to emotion regulation strategies commonly present in alexithymic individuals [113]. The similarity of content and underlying processes of difficulty in identifying feelings (a dimension of alexithymia) and lack of emotional awareness (a dimension of emotion regulation difficulties) forms a common substrate for both constructs and may be a region of overlap. The observed emotion regulation difficulties in alexithymic individuals appear to be conceptually attributed to a lack of emotional awareness and differentiation [118]. 

Consistent with results from other studies, it was predominantly the difficulties with identifying feelings (DIF) aspect of alexithymia that was related to aggression [119,120,121]. Additionally, DIF significantly predicted total BAQ and anger and mediated the relationship between hostility and anger. In other words, the relationship between the cognitive and the affective components of aggression is mediated by the difficulties with identifying feelings dimension of alexithymia. Among those, having difficulty in identifying their feelings of hostile aggression motivated by anger [122] may be considered as a further effort to distract from feelings (or to express feelings in a rather maladaptive way).

Conscious awareness of emotions is acquired when emotional information and experiences are integrated into cognitive processes [123]. Furthermore, it has been argued that many symptoms typical of schizophrenia can be explained by specific cognitive deficits in schizophrenic patients to accurately attribute mental states to themselves or others [124]. Affective theory of mind refers to the ability to comprehend and confront others’ affective mental states and differences in “affective” versus “non-affective” theory of mind’ tasks have been reported to relate to certain behaviors in schizophrenia, such as violence [125].

The drastic changes in the subjective experiences of schizophrenic patients give rise to stress and bring about everyday challenges. The ability to manage stressful situations, mental resilience and sense of coherence predict and modify the level of psychological wellbeing among patients suffering from mental disorders [69]. The theory of salutogenesis supports the idea that sense of coherence mitigates stress, and results from early studies evidence the protective effects of a salutogenic approach in individuals with serious mental illnesses [126].

The first step when dealing with a stressful situation is to perceive and be knowledgeably aware of its full extent [53]. An individual with higher comprehensibility is more likely to perceive stimuli from the environment as coherent and understandable [127]. Schizophrenic patients usually require support in reassembling their erratic experiences, reflecting upon them and possibly learning something from them. The severity of symptoms and the chronicity of the psychiatric disorder seem to determine their ability to adapt, as does the use of available resources to manage stressors. First-episode psychotic patients in remission appear to be in a more favorable position compared to chronic patients with persistent positive symptoms or in a deficit state [128,129]. 

In mental health settings where the therapeutic treatment and long-term follow-up of psychiatric patients is sought, the theory of salutogenesis may find practical application through the modification of therapeutic procedures and environmental factors in order to enhance the three components of salutogenesis [130]. The same approach could even be applied in clinical practice in situations involving conflict prevention through communication, emotional regulation, self-management and other de-escalation techniques [131]. The first goal attempted in de-escalation is reducing the patient’s level of arousal to enable discussion by gathering the necessary resources through manageability. The patient should be encouraged to communicate openly with staff about their own emotions and to discuss feelings of anger and frustration in order to enhance comprehensibility. Recent evidence supports the effectiveness of integrating milieu therapy in psychiatric acute wards in reducing conflict behaviors among schizophrenia patients [132]. 

Preventing or managing stress is of paramount importance according to the diathesis–stress model of schizophrenia. Considering that sense of coherence essentially protects against the pressures generated by stressors, should clearly define the purpose of implemented healthcare interventions, in order to avoid negative health outcomes. Since patients with schizophrenia are so vulnerable to the impacts of stressors, the focus of comprehensive psychoeducation should be on reinforcing coping skills by teaching stress reduction techniques and assertiveness training. Additionally, providing schizophrenic patients with education regarding their illness and improving attention along with other cognitive functions would enhance comprehensibility. When facing challenges, equally important for these patients is acknowledging, approaching, and activating or resorting to other available resources, namely family, social and healthcare support. 

In summary, it is reasonable to assume that the inability to recognize and describe emotions may, under stressful circumstances, lead to an increased state of unmanageable arousal. Patients with schizophrenia, particularly if they are characterized by temperamental hostility, feel threatened under stressful conditions and tend to react aggressively. Furthermore, in individuals with reduced abilities to regulate emotions with conscious effort or a strong tendency toward impulsivity, the effect is reinforcing. In these cases, the explanation for the apparent aggressive behavior is provided by the indirect effect of alexithymia, which reduces the cognitive and emotional capacities necessary to moderate distressing feelings and inhibit impulsive actions.

Specific therapeutic interventions to improve the ability to identify subjective feelings that target alexithymia and neurocognitive impairments that may make self-reflection difficult could be implemented. Integrative psychotherapy that targets metacognition could assist patients in developing these capacities [133], and cognitive remediation [134] could provide the prerequisites for metacognitively focused psychotherapy to be successful. Additionally, research argues that alexithymic individuals would benefit more from group-based psychological therapy and supportive and educational approaches as opposed to interpretive ones [25]. A recent open-label randomized controlled trial applied an integrated cognitive-based violence intervention program on management of repetitive violence in patients with schizophrenia and evidenced significant improvement of cognitive failure, management of alexithymic features and attribution styles and errors and fostered adequate decision-making styles and emotion regulation capacity. This intervention provided patients a more active role to manage their violent behavior with the involvement of alexithymia [75].

In the last two decades, there has been increasing research interest in the ability of tailored interventions to modify and strengthen the sense of coherence of various target groups [97]. Enhancing comprehensibility for individuals with schizophrenia should be the focus of comprehensive psychoeducation and cognitive remediation. Moreover, aiming to improve prognosis through the implementation of psychosocial rehabilitation interventions requires the identification and reduction of aggravating factors, such as ineffective stress management, as well as the enhancement of protective factors, especially manageability.

Aggressive behavior is a leading public health problem incurring a massive cost on society and, according to epidemiological studies, individuals diagnosed with major mental disorders such as schizophrenia are more likely to be engaged in such behaviors than the general population, with obvious relevance for health care systems. The etiological heterogeneity of aggression and the possible multifactorial contributors to aggressive behaviors, along with the fact that the current treatment approaches and outcomes are so far inferior, justifies the search for new targets for the treatment of aggression in people with schizophrenia [135]. Addressing factors that limit the effectiveness of treatments might decrease the burden of these severe chronic disorders. Given the high observed prevalence of alexithymia and associated negative outcomes, researchers and clinicians examined the feasibility of treating alexithymia [136]. Interventions aiming to increase patients’ emotional awareness and their ability to label emotions may promote their successful engagement in cognitive behavioral psychotherapies to regulate unpleasant, angry emotions before they escalate and drive their behavior [137]. There is promising evidence that alexithymia can improve with treatment even after neurological damage [32].

Identified targets for treating aggression in schizophrenia in our study, i.e., alexithymia and sense of coherence, are mostly amenable to psychotherapeutic and psychosocial interventions. Several different psychotherapeutic approaches for schizophrenia have been developed and studied, with cognitive behavior therapy having the strongest evidence base [138]. Providing comprehensive psychological interventions in this clinical population will likely require drawing upon knowledge from several areas of current research and incorporating elements of various psychosocial interventions, such as cognitive remediation, social skills training and psychoeducation [139]. A recent systematic review evidenced the effectiveness of cognitive remediation and social cognitive training in the management of violent and aggressive behaviors in schizophrenia [140]. According to another review, once patients’ positive symptoms have stabilized, cognitive behavioral therapy and cognitive remediation are the two psychosocial interventions that have demonstrated positive outcomes for violence in patients with schizophrenia [141]. 

The presence of contributing factors to aggression, either adverse or protective, namely alexithymia and sense of coherence, acquires importance in light of the possibility of handling their impact on the effectiveness of antiaggressive treatments, particularly in the domain of psychosocial interventions. Testing the efficacy of psychotherapeutic interventions for people with schizophrenia may benefit from an inclusion of aggression as a treatment outcome in clinical trials. Our study proposes alexithymia and sense of coherence as putative targets for addressing aggression in schizophrenia and extends ideas for treatment and research. Future studies should be carried out, especially controlled and follow-up studies, comparing different forms of treatment on more extensive patient populations while considering potential confounding factors and performing objective assessment of aggression, alexithymia and related constructs.

The present study suffers from various limitations. Participants were enrolled from the outpatients department. Subsequently, research findings may not be applied beyond this study population. Additionally, since the data were collected using self-report tools, self-serving bias may be an issue. Specifically, the TAS-20 scale as a measure of alexithymia consists of self-rated agreement statements and presupposes awareness of the deficit to be reported, which raises the concern that emotion recognition deficits that patients do not detect would not be captured by this scale. Moreover, due to social desirability bias, participants might have stated their self-reported levels of aggression in a socially acceptable manner instead of providing answers that are reflective of their genuine aggression level. Finally, the cross-sectional design of the study precluded us from making secure inferences about direction of causality.

## 5. Conclusions

High rates of aggression and alexithymia, along with low sense of coherence, were observed among schizophrenic outpatients. Alexithymia fueled aggression, and sense of coherence counteracted aggressive tendencies. The difficulty identifying feelings dimension of alexithymia emerged as a liability, and the comprehensibility component of sense of coherence as a protective factor buffering the deleterious consequences towards physical aggression. These results hold practical implications for the treatment and rehabilitation of schizophrenic patients. 

## Figures and Tables

**Table 1:** General characteristics of participants and scores on BAQ, TAS, SOC and subscales.

	N	Min	Max	Mean	SD
**age**	100	21	65	41.71	10.718
**education**	98	6	18	13.10	3.003
**BAQ**	100	14	54	30.93	8.247
**TAS**	100	20	82	49.10	13.369
**SOC**	99	19	89	56.57	15.697
**PA**	100	3	15	6.61	3.284
**VA**	100	4	15	10.06	2.473
**H**	100	3	15	8.24	3.194
**A**	100	3	13	6.02	2.655
**DIF**	100	7	31	16.91	6.741
**DDF**	100	5	25	13.07	4.965
**EOT**	100	8	36	19.12	5.524
**SOC A**	99	4	28	18.82	5.530
**SOC B**	99	5	35	21.39	7.184
**SOC C**	99	4	28	16.35	5.877

Abbreviations: P, participants; D.S., descriptive statistics; PA, physical aggression; VA, verbal aggression; H, hostility; A, anger; DIF, difficulty identifying feelings; DDF, difficulty describing feelings; EOT, externally oriented thinking; SOC A; meaningfulness; SOC B, comprehensibility; SOC C, manageability.

**Table 2:** Correlations among age, education (in years), TAS, BAQ and SOC.

Pearson Correlation N = 100		AGE	Education (in Years)	Sense of Coherence (SOC)	Toronto Alexithymia Scale (TAS)
Education (in years)	r	0.073			
	*p*	0.478			
Sense of coherence (SOC)	r	−0.021	0.003		
	*p*	0.838	0.976		
Toronto Alexithymia Scale (TAS)	r	0.044	−0.062	−0.624 **	
	*p*	0.664	0.545	0.000	
Brief aggression questionnaire (BAQ)	r	0.058	0.057	−0.653 **	0.525 **
	*p*	0.569	0.575	0.000	0.000

** *p* < 0.01.

**Table 3:** Correlations among subscales of TAS, BAQ and SOC.

N = 100		DIF	DDF	EOT	TAS	SOC A	SOC B	SOC C	SOC
**BAQ**	r	0.579 **	0.463 **	0.147	0.525 **	−0.366 **	−0.648 **	−0.608 **	−0.525 **
	*p*	0.000	0.000	0.144	0.000	0.000	0.000	0.000	0.000
**PA**	r	0.435 **	0.384 **	0.163	0.429 **	−0.298 **	−0.487 **	−0.419 **	−0.485
	*p*	0.000	0.000	0.105	0.000	0.003	0.000	0.000	0.000
**VA**	r	0.140	−0.127	−0.004	0.116	0.068	−0.261 **	−0.310 **	−0.212 *
	*p*	0.166	0.207	0.967	0.251	0.506	0.009	0.002	0.035
**H**	r	0.444 **	0.292 **	0.151	0.394 **	−0.328 **	−0.534 **	−0.456 **	−0.531 **
	*p*	0.000	0.003	0.135	0.000	0.001	0.000	0.000	0.000
**A**	r	0.595 **	0.495 **	0.078	0.516 **	−0.433 **	−0.517 **	−0.526 **	−0.586 **
	*p*	0.000	0.000	0.442	0.000	0.000	0.000	0.000	0.000

Abbreviations: PA, physical aggression; VA, verbal aggression; H, hostility; A, anger; DIF, difficulty identifying feelings; DDF, difficulty describing feelings; EOT, externally oriented thinking; SOC A; meaningfulness; SOC B, comprehensibility; SOC C, manageability. * *p* < 0.05 or ** *p* < 0.01.

**Table 4:** Summary of hierarchical regression analysis for variables predicting aggression (BAQ scores).

		Unstandardized Coefficients		Standardized Coefficients				
		B	Std. Error	Beta	t	Sig.	R Square	∆R ^2^
**Step 1**	**(constant)**	26.906	4.740		5.676	0.000	**0.009**	**0.009**
	**age**	0.053	0.080	0.072	0.660	0.511		
	**gender**	−0.533	1.771	−0.034	−0.301	0.764		
	**education**	0.170	0.278	0.065	0.613	0.542		
**Step 2**	**(constant)**	11.954	4.850		2.465	0.016	**0.270**	**0.261**
	**age**	0.046	0.069	0.063	0.666	0.507		
	**gender**	−1.183	1.532	−0.075	−0.772	0.442		
	**education**	0.273	0.241	0.104	1.135	0.259		
	**TAS**	0.306	0.053	0.514	5.737	0.000 *		
**Step 3**	**(constant)**	37.840	6.391		5.921	0.000	**0.448**	**0.178**
	**age**	0.048	0.060	0.066	0.800	0.426		
	**gender**	−1.410	1.340	−0.089	−1.052	0.296		
	**education**	0.235	0.211	0.089	1.117	0.267		
	**TAS**	0.108	0.059	0.180	1.811	0.073		
	**SOC**	−0.272	0.050	−0.538	−5.414	0.000 *		

Correlations are statistically significant at the * *p* < 0.01 level. Beta = standardized regression coefficient.

**Table 5:** Hierarchical regression analyses for variables (TAS and SOC subscales) predicting aggression (BAQ and subscales).

Dependent Variables		Unstandardized Coefficients		Standardized Coefficients				
		B	Std. Error	Beta	t	*p*	R Square	∆R ^2^
**BAQ**	**DIF**	0.287	0.146	0.232	1.967	0.05 *	33.4	33.4
	**SOC B**	−0.415	0.136	−0.363	−3.045	0.003 **	49.9	16.5
	**SOC C**	−0.349	0.149	−0.250	−2.340	0.021 *		
**PA**	**SOC B**	−0.134	0.066	−0.291	−2.041	0.044 *	27.9	7.4
**VA**	**SOC A**	0.142	0.044	0.319	2.609	0.011 *	17.1	15.3
	**SOC C**	−0.118	0.058	−0.281	−2.051	0.043 *		
**H**	**SOC B**	−0.174	0.062	−0.392	−2.833	0.006 **	32.5	13.2
**A**	**DIF**	0.117	0.050	0.294	2.335	0.022 *	42.7	5.9

Correlations are statistically significant at the * *p* < 0.05 or ** *p* < 0.01 level. Abbreviations: PA, physical aggression; VA, verbal aggression; H, hostility; A, anger; DIF, difficulty identifying feelings; SOC A; meaningfulness; SOC B, comprehensibility; SOC C, manageability. Notes: 1. Results are given for Step 2, when SOC variables are included as independent variables. 2. Only the variables that significantly predicted the dependent variables are shown.

**Table 6:** Mediation analysis of sense of coherence (SOC) on Toronto alexithymia scale (TAS) – brief aggression questionnaire (BAQ) relationship.

Variable	b	SE	t	*p*	95% Confidence Interval	
					LLCI	ULCI
TAS → SOC	−0.7367	0.0936	−7.8672	0.000	−0.9225	−0.5508
TAS → BAQ	0.3193	0.0538	5.9358	0.000	0.2125	0.4260
TAS → SOC → BAQ	−0.2841	0.0510	−5.5764	0.000	−0.3853	−0.1830
Effects						
Direct	0.1100	0.0601	1.8285	0.0706	−0.0094	0.2293
Indirect *	0.2093	0.0445			0.1234	0.2966
Total	0.3193	0.0538	5.9358	0.000	0.2125	0.4260

* Based on 5000 bootstrap samples.

**Table 7:** Mediation analysis of difficulty identifying feelings (DIF) on hostility (H) – anger (A) relationship.

Variable	b	SE	t	*p*	95% Confidence Interval	
					LLCI	ULCI
H → DIF	0.9366	0.1910	4.9033	0.0000	0.5575	1.3156
H → A	0.3202	0.0775	4.1331	0.0001	0.1665	0.4740
H → DIF → A	0.2080	0.0354	5.8810	0.0000	0.1378	0.2782
Effects						
Direct	0.1255	0.0746	1.6814	0.0959	−0.0226	0.2736
Indirect *	0.1948	0.0493			0.1041	0.2975
Total	0.3202	0.0775	4.1331	0.0001	0.1665	0.4740

* Based on 5000 bootstrap samples. The model explains 60.8% of the variance in the outcome variable.

**Table 8:** Mediation analysis of comprehensibility (SOC B) on hostility (H)–physical aggression (PA) relationship.

Variable	b	SE	t	*p*	95% Confidence Interval	
					LLCI	ULCI
H → SOC B	−1.2002	0.1928	−6.2264	0.0000	−1.5828	−0.8176
H → PA	0.5052	0.0913	5.5346	0.0000	0.3241	0.6863
H → SOC B → PA	−0.1446	0.0460	−3.1416	0.0022	−0.2359	−0.0532
Effects						
Direct	0.3317	0.1033	3.2095	0.0018	0.1266	0.5368
Indirect *	0.1735	0.0657			0.0539	0.3085
Total	0.5052	0.0913	5.5346	0.0000	0.3241	0.6863

* Based on 5000 bootstrap samples. The model explains 34.34% of the variance in the outcome variable.

**Table 9:** Mediation analysis of manageability (SOC C) on anger (A)–physical aggression (PA) relationship.

Variable	b	SE	t	*p*	95% Confidence Interval	
					LLCI	ULCI
A → SOC C	−1.1650	0.1914	−6.0850	0.0000	−1.5449	−0.7850
A → PA	0.4552	0.1175	3.8728	0.0002	0.2219	0.6884
A → SOC C → PA	−0.1758	0.0600	−2.9278	0.0043	−0.2949	−0.0566
Effects						
Direct	0.2504	0.1331	1.8820	0.0626	−0.0137	0.5145
Indirect *	0.2048	0.0771			0.0594	0.3617
Total	0.4552	0.1175	3.8728	0.0002	0.2219	0.6884

* Based on 5000 bootstrap samples. The model explains 45% of the variance in the outcome variable.

## Data Availability

The data and the questionnaires of the study are available upon request from the corresponding author.
